# Pure Stimulants

**Published:** 1869-08

**Authors:** 


### Pure Stimulants.

We have received, from F. Briggs & Co., specimens of Old Rye, Monongahela and Bourbon Whiskies, which appear to us as very pure and fine. Those who desire stimulants for medicinal purposes, will do well to call upon this firm, corner Washington and Quay Streets, where will be found a general assortment of the choicest American and imported liquors.

